# Advances in Bovine Coronavirus Epidemiology

**DOI:** 10.3390/v14051109

**Published:** 2022-05-21

**Authors:** Qinghe Zhu, Bin Li, Dongbo Sun

**Affiliations:** 1 Heilongjiang Provincial Key Laboratory of the Prevention and Control of Bovine Diseases, College of Animal Science and Veterinary Medicine, Heilongjiang Bayi Agricultural University, No. 5 Xinfeng Road, Sartu District, Daqing 163319, China; qinghezhumy@126.com; 2 Institute of Veterinary Medicine, Jiangsu Academy of Agricultural Sciences, Key Laboratory of Veterinary Biological Engineering and Technology, Ministry of Agriculture, Nanjing 210014, China

**Keywords:** bovine coronavirus, epidemiology, genetic evolution, cross-species transmission

## Abstract

Bovine coronavirus (BCoV) is a causative agent of enteric and respiratory disease in cattle. BCoV has also been reported to cause a variety of animal diseases and is closely related to human coronaviruses, which has attracted extensive attention from both cattle farmers and researchers. However, there are few comprehensive epidemiological reviews, and key information regarding the effect of S-gene differences on tissue tendency and potential cross-species transmission remain unclear. In this review, we summarize BCoV epidemiology, including the transmission, infection-associated factors, co-infection, pathogenicity, genetic evolution, and potential cross-species transmission. Furthermore, the potential two-receptor binding motif system for BCoV entry and the association between BCoV and SARS-CoV-2 are also discussed in this review. Our aim is to provide valuable information for the prevention and treatment of BCoV infection throughout the world.

## 1. Introduction

Bovine coronavirus (BCoV) is a single-stranded positive-sense RNA virus with a lipid envelope belonging to the order *Nidovirales*, family *Coronaviridae*, subfamily *Orthocoronavirinae*, genus *Betacoronavirus*, and subgenus *Embecovirus*. The genus *Betacoronavirus* is also important for humans as it includes severe acute respiratory syndrome-related coronavirus, Middle-East respiratory syndrome-related coronavirus, and severe acute respiratory syndrome coronavirus 2 (SARS-CoV-2) [1]. BCoVs are widespread throughout the world due to rapid viral transmission via the fecal–oral and respiratory routes, as well as the existence of carrier animals within infected herds. BCoV is responsible for significant economic losses due to the high mortality of calves, reduced growth performance in feedlot cattle, and decreased milk production of adult dairy cattle [2]. In addition, different bovine-like coronaviruses have been identified as the potential etiologic pathogens of enteric and/or respiratory diseases in a diverse spectrum of ruminant species, dogs, and even humans, suggesting possible cross-species viral transmission [2,3,4]. Unfortunately, there are few comprehensive reviews on BCoV origin, epidemiology, and co-infections with other intestinal and respiratory pathogens of BCoV. BCoV exhibits the tissue tropism for both the intestine and respiratory tract, and can cause serious damage to both organs. However, the key information regarding the Spike (S)-gene differences between intestinal and respiratory BCoV strains remains unclear. Although BCoV may be of great significance to the field of cattle industry and even human biosafety, a large number of research challenges must be overcome. Therefore, this study reviews the epidemiology, genetic evolution, potential cross-species transmission, and variability in the S genes of both intestinal and respiratory BCoV strains. Finally, this review proposes future prospects. Hopefully, the review can provide valuable insights for further research on BCoV.

## 2. The Prevalence of BCoV

### 2.1. The Origin of BCoV

**Figure 1:**
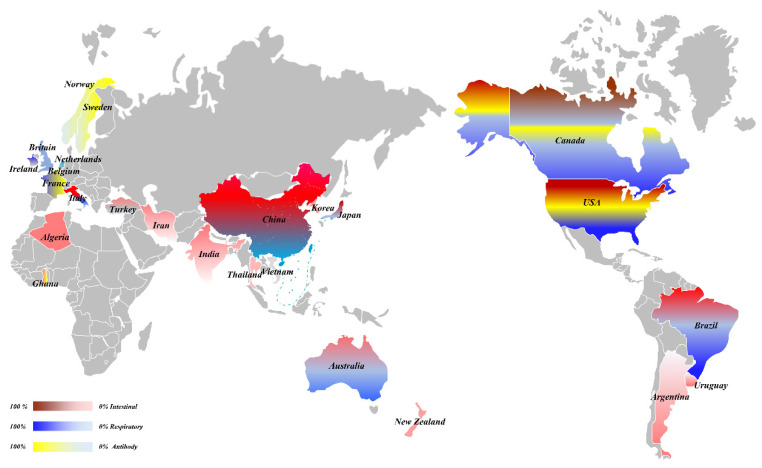
The overall prevalence of BCoV.

In 1972, Mebus et al. was the first to identify a virus that could cause severe diarrhea in calves, and subsequently identified the virus as a coronavirus via histology, immunofluorescence, and immunoelectron microscopy [5]. However, only enteric histology was observed and lung-tissue injury was not analyzed. Further progress was made in 1984, when McNulty et al. (1984) isolated BCoV from the lung of a calf suffering from bronchopneumonia. Challenge with BCoV produced a mild clinical disease with upper respiratory tract infection as the main clinical feature. Therefore, it was determined that BCoV could cause respiratory tract and diarrhea symptoms in cattle [6]. Subsequently, the diarrhea and respiratory symptoms caused by BCoV have been widely reported in the Americas, Europe, Asia, Oceania, and even Africa (Figure 1) [2,7,8,9,10,11]. Therefore, a comprehensive study on the epidemiology, diagnosis, and vaccine development of BCoV has been performed all over the world [12,13,14,15,16,17,18].

### 2.2. The Transmission of BCoV

#### 2.2.1. The Transmission of Enteric BCoV

Since BCoV was first detected in the United States, it has since been reported in five continents of the world; however, the incidence rate and the time of occurrence caused by BCoV infection varies between countries (Table 1). Prior to 2000, BCoV was reported to cause intestinal symptoms in the Americas, Europe, and Asia. The positive rates in the USA, Canada, and Argentina in the Americas were higher, reaching 2.41–84% [9,19,20,21,22,23]. The positive rates in Britain (1986) and Belgium (1999) in Europe were 14% and 8% [24,25], respectively. From 2000 to 2009, the BCoV positive rates for Turkey and Korea in Asia were 10.8–28.1% and 5.6–58.2%, respectively [26,27,28,29,30,31,32], and the positive rates of the Netherlands and Italy in Europe were 2.80% and 46.74%, respectively [7,33]; whereas Brazil in South America had a high positive rate of 68.6% [34,35,36,37]. From 2010 to 2019, BCoV started to appear and spread throughout Oceania. The positive rates of BCoV in fecal samples from Australia and New Zealand were 21.6% and 14.0%, respectively [38,39]. At the same time, the virus also appeared in Africa. The positive rates in stool samples from Algeria and Ghana were 20.73% and 0.30%, respectively [8,40]. In addition, BCoV outbreaks in Asia were reported during the same period. BCoV was detected in diarrhea stool samples in many countries, including Iran, China, Thailand, India, and Vietnam, with positive rates of 7.2%, 12.20–69.05%, 12%, 8.88–16.00%, and 6.9%, respectively [11,41,42,43,44,45]. It can be observed that BCoV was first identified in the Americas, and then successively appeared in Asia, Europe, Oceania, and Africa. In particular, after 2010, BCoV exhibited an epidemic trend in many countries on five continents and caused intestinal manifestations of diarrhea.

#### 2.2.2. The Transmission of Respiratory BCoV

BCoV was first detected in respiratory samples from the USA [1]. In 1999, positive results were also reported in nasal-swab samples from Japan in Asiae [10]. From 2000 to 2009, the positive rates in Italy and Ireland were 9.60–65.85% and 22.9–60.7%, respectively [50,51]. From 2010 to 2019, Australia in Oceania detected BCoV in respiratory samples for the first time, with a positive rate of 13.00–33.33% [55]. After 2020, BCoV was detected in respiratory samples for the first time in China, with a positive rate of 21.53% [45]. To date, there have been no reports of BCoV-positive respiratory samples in Africa.

#### 2.2.3. The Serological Surveys of BCoV

Serological surveys and analysis showed that the positive rates of serum antibodies in various countries differed, but the positive rate was generally very high. In Canada and Sweden, the highest positive rate was 100% [56,57], whereas the positive rates of the USA, Norway, France, and Belgium were 11–91%, 16.0–72.2%, 16.5%, and 30%, respectively [22,58,59,60,61,62,63]. Although the positive rate of pathogen detection in Ghana was only 0.3%, the positive rate of serum antibodies reached 55.8% [8].

### 2.3. The BCoV Infection-Associated Factors

According to references, BCoV can cause diarrhea in cattle at any time of the year; however, the frequency of BCoV outbreaks is reportedly higher during colder months than in warmer months [4,18,19,20,21,22,23]. The overall incidence rate of BCoV during the cold season is approximately 11.8–60.97%, whereas that in the warm season is about 1.5–48.83% [18,23,24]. The detection of BCoV in respiratory samples also exhibited similar results. Although BCoV was detected in calf respiratory submissions throughout the year, detection rates peaked in early winter (44.1%) and remained elevated until the early summer months (9.3%) [6].

Some studies have demonstrated that age was significantly associated with a positive rate of BCoV. According to the previous reports, the BCoV infection rates of calves aged 1–5 weeks were 6.50–34.61%, 5.50–38.46%, 6.25–20.90%, 16.40–44.44%, and 5.90–32.92%, respectively [31,39,40,64,65]. Further analysis revealed that the infection rate in the 4-week-old group was highest (44.44%) [40], and this age group was considered to be the most susceptible age group to BCoV infections. In addition, the incidence of diarrhea in calves was high, whereas respiratory symptoms in adult cattle were more common [2,21,29,66]. Moreover, respiratory BCoV infection in adult cattle may be more fatal [2,33,50].

Different breeds of cattle can be infected with BCoV. The study by Bok et al. (2015) found that the infection rate of BCoV in dairy cattle was significantly higher than that in beef cattle, with infection rates of 5.95% (63/1058) and 1.71% (92/5365), respectively [20]. The higher incidence rate of disease in cows may be related to the frequent exposure to farms, including the sharing of equipment and movement of people and vehicles between farms, all of which may play a major role in infectious disease transmission [26]. In addition, reports have shown that BCoV infection in the respiratory tract of beef cattle occurred relatively frequently [23,37,58,67,68]. This effect may be attributed to beef cattle being more involved in transportation; transport stress is typically one of the main causes of bovine respiratory disease syndrome [69,70].

Statistical analysis of the presence of BCoV in the feces of healthy calves and those with diarrhea showed that the detection rate of BCoV in healthy calves was between 0% and 46%, and that diarrhea in calves was between 3.4% and 69.0% [19,39,65,71]. In general, the detection rate of BCoV in calves with diarrhea was higher than that in the healthy calves.

### 2.4. Co-infection of BCoV with Typical Bovine Enteric and Respiratory Pathogens

#### 2.4.1. Co-infection of BCoV with Typical Bovine Enteric Pathogens

BCoV can co-infect with a variety of enteric pathogens, including parasites, bacteria, and viruses, resulting in diarrhea. Of these, co-infection with *Escherichia coli* was found to be the most common, with a positive rate of 0.7–36.84%. Co-infection with rotavirus was the main cause of viral diarrhea in calves, with a co-infection rate of 2.43–13.15%. The results of an investigation into co-infected parasites revealed that the incidence of diarrhea caused by BCoV and *Eimeria* co-infection was 1.49% and co-infection with *Cryptosporidium* was 5.4% [19,22,25].

#### 2.4.2. Co-infection of BCoV with Typical Bovine Respiratory Pathogens

BCoV can establish a co-infection with a variety of respiratory pathogens and cause respiratory symptoms [52,72]. O’Neill et al. (2014) found that BCoV represented the most frequently detected partner virus in bovine respiratory diseases, accounting for 60.7% of all multi-virus detection. Among them, the co-infection rate of BCoV and parainfluenza virus-3 was 18.8%, the co-infection rate with bovine herpesvirus-1 was 9.1%, and the co-infection rate with BVDV was 9.1% [52,73]. The co-infection rate with bovine respiratory syncytial virus (BRSV) was the highest, with could reach as high as 23.7% [64,74,75]. Therefore, BCoV and BRSV may represent the most frequent pairing, and deserve particular attention in terms of farm management and research. In addition, in the cases of respiratory diseases caused by bacterial co-infection, the co-infection rate of BCoV and *Pasteurella* reached 68% (17/25) [70,76]. One possible explanation of this high co-infection rate is that BCoV can enhance bacterial adherence by upregulating the expression of cellular receptors on bovine respiratory epithelial cells [77].

### 2.5. Pathogenicity of BCoV

The results of BCoV challenge experiments have shown that BCoV can cause severe diarrhea in calves. Some calves also manifest respiratory symptoms after diarrhea. Upon histopathological examination, the BCoV-inoculated calves exhibited mild to severe villous atrophy, widespread villous fusion, and increased crypt depth in the small intestine. The epithelia of the alveoli, bronchi, and bronchioles often appeared desquamated or necrotic. In addition, BCoV RNA was transiently detected in the serum samples of the calves, revealing that oral infection leads to viremia [66,78].

Other animal pathogenicity tests revealed that turkeys developed clinical symptoms 72 h after BCoV inoculation, and coronavirus particles were detected by electron microscopy from the enteric contents of turkeys [79]. Kaneshima et al. (2007) orally inoculated three 1-month-old pups with BCoV, and housed them together with a non-inoculated control group. The results confirmed the presence of BCoV antibodies in both the challenge and control groups, and despite the detection of BCoV genes in the oral and rectal swabs by RT-PCR, no respiratory symptoms or diarrhea were observed [80].

The pathogenicity analysis showed that BCoV could cause different degrees of respiratory and enteric injury in cattle, but exhibited stronger enteric tropism. In other animals, BCoV is pathogenic to turkeys and pups, and BCoV can be transmitted between pups; however, the main manifestation remained enteric symptoms or asymptomatic.

Previous reports showed that BCoV shares a high nucleotide and antigenic similarity with canine respiratory coronavirus (CRCoV) and Turkey coronavirus (TCV), respectively [79,80]. These reasons prompted researchers to initiate other animal pathogenicity study. Recent studies revealed that CRCoV and BCoV share receptor specificity, utilizing sialic acids for cell surface attachment, internalization, and entry, and they appear to employ human leukocyte antigen class I (HLA-1) as the entry receptor [81]. Hence, the viral receptor sequences analysis of CRCoV and BCoV S proteins may be helpful to explore the key sites of cross-species transmission.

## 3. Genetic Evolution of BCoV

### 3.1. Phylogenetic Analyses of BCoV Strains

**Figure 2:**
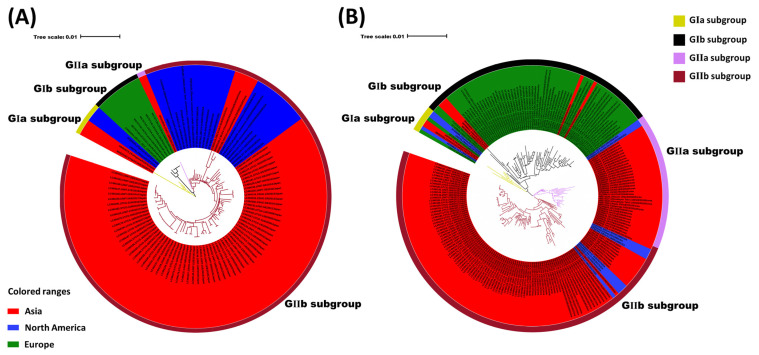
Phylogenetic analysis of BCoV strains. (**A**) Phylogenetic analysis of the global BCoV strains based on the whole genome. (**B**) Phylogenetic analysis of the global BCoV strains based on the S gene. Strain name, source: enteric (E), respiratory (R), and unknown (U), year of collection, country, and the GenBank accession number are indicated.

Molecular clock analyses estimated that the BCoV ancestor emerged in the 1940s [52] and the virus may have been derived from recombination events similar to that of SARS [82]. The global BCoV reference strains (Table S1) were primarily divided into European (GI) and Asian-American (GII) groups. Although there are some differences between the early Asian-American and the European types [52], the increase in epidemiological data (i.e., S gene and whole genome research), the genetic diversity of the strains has gradually been revealed. In particular, phylogenetic analyses have shown that several classical original strains are clustered together, the European strains are clustered together, and American and Asian strains are clustered into a large category (Figure 2A). According to the phylogenetic tree construction analysis based on the S gene, BCoV can be primarily divided into GI and GII groups. Through further divisions, it was found that early classical strains from various countries clustered to form the GIa subgroup, including some strains from Asia, America, and Europe, and the original Mebus strains. The BCoV strains from Europe formed the GIb subgroup. Most of the BCoV strains from America and Asia clustered to form the GII group. The phylogenetic-tree analysis also showed that the Korean BCoV strains independently formed the GIIa subgroup. Some BCoV strains from America, Japan, and Vietnam, together with BCoV strains from China, formed the GIIb subgroup (Figure 2B). The results further demonstrate the geographical clustering of BCoV, in which the Asian BCoV strains were closely related to the American BCoV strains, and European BCoV strains were clustered independently. This finding may be related to the frequent trade between America and Asia [10,14]. The phylogenetic tree showed no obvious clustering between the S genes of respiratory BCoV (RBCoV) and enteric BCoV (EBCoV), which was similar to that of the previous studies [83]. In addition, the phylogenetic analysis demonstrated the clustering of BCoVs/bovine-like coronaviruses according to the year or country of detection/isolation, suggesting a likely co-evolution with continuous exchange by the respective virus pools.

**Figure 3:**
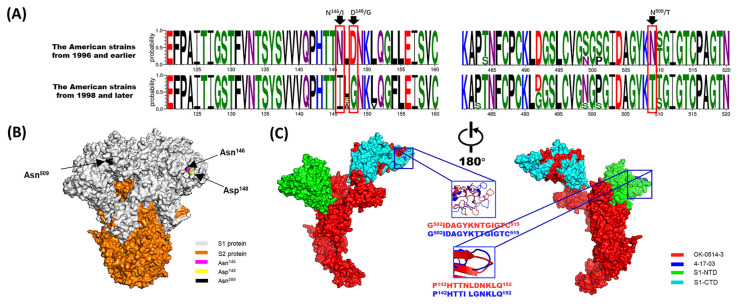
Analysis of amino acid mutations in the BCoV S protein. (**A**) Divergence analysis of the S proteins between the American strains from 1996 and earlier and from 1998 and later. (**B**) A predicted 3D structural model of the OK-0514-3 strain S protein was generated and observed on the front sides. Note: The S1 and S2 proteins are colored gray and orange, respectively. Amino acids 146, 148, and 509 are colored magenta, yellow, and black, respectively. (**C**) A comparative analysis of the predicted S-protein modeling of the OK-0514-3 and 4-17-03 (representative strain of the American strains from 1996 and earlier and from 1998 and later, respectively). Note: The OK-0514-3 strain is shown as the surface and in red in the illustration. The mutant amino acid residues of the S protein of the 4-17-03 strain are shown as the surface and in blue in the illustration. The N-terminal domain of the S1 subunit (S1-NTD: residues 15–298) and C-terminal domain of the S1 subunit (S1-CTD: residues 326–540) proteins are shown as the surface in green and cyan, respectively.

In addition, some studies have identified the USA as the only source of introduction of BCoV to other countries [84]. Our study found that American BCoV strains were distributed throughout all subgroups. Therefore, the evolution of American BCoV strains may represent the evolution of global strains to a certain extent. The analysis of BCoV S protein showed that the amino acid sites 146, 148, and 509 were gradually transformed into 146N–146I, 148D–148G, and 509N–509T, respectively, over time during virus evolution, resulting in structural changes (Figure 3). The amino acid sites 146 and 148 were located in the NTD and near the sialic acid-binding sites [85]. Thus, mutations may affect the glycans binding to the NTD. In addition, it was previously reported that amino acid 509 was located at the CTD (putative receptor-binding domain) and also functioned as the binding site of positive pressure selection of the S gene. The changes in these three amino acid sites occurred in the American strains from 1996 to 1998, which suggested that BCoV may have undergone taxonomic evolution around the 1990s. Although the mutations occurred in the American BCoV strains, the mutations corresponded to the GI GII groups. Therefore, these mutation sites may also represent important marker sites for the differentiation of the GI and GII groups. Moreover, it has been reported that amino acid sites 146 and 148 on the S gene may differ between respiratory and enteric strains [86]. Therefore, the evolutionary relationship of the American strains may also be of great significance for the study of the evolutionary characteristics associated with intestinal and respiratory tropism.

### 3.2. Comparison Analysis of BCoV Strains between the Enteric and Respiratory Tracts

To date, the determinants of tropism in EBCoV and RBCoV remain controversial. The S gene mediates virus entry and various coronavirus studies have also confirmed that variation of the S gene leads to a change in tissue tropism [26]. Therefore, tropism research has focused on the difference in the S gene. According to the results of the previous studies, sites 113, 115, 146, 148, 501, 646, 510, and 531 amino acids on the S gene may represent different sites between the RBCoV and EBCoV strains [2,3,9,27,28,29]. In this study, no clear clustering was observed between the S genes of respiratory and enteric BCoV based on the phylogenetic tree; however, geographic clustering was clearly observed. Therefore, we conducted a comparative analysis of the RBCoV and EBCoV strains from the same country with a similar separation time. Alignment analysis of the amino acid sequences of the S genes from the 6 BCoV strains identified from the nasal swab samples and 20 BCoV strains identified from the fecal samples in Sweden, showed that there were differences in the dominant amino acids of the five amino acid sites, T113(4/6)-I113(20/21), D115(4/6)-I115(19/20), I447(5/6)-T447(19/20), Y471(4/6)-H471(19/20), and N510(4/6)-S510(18/20). However, no significant differences were found in the comparison of respiratory and enteric BCoV for other countries. Some studies support the hypothesis that over time, BCoV evolved from being solely enteric to a dual enteric and respiratory tropic virus [30]. Therefore, the study of the relationship between differential amino acids and tissue tropism may require further analysis of the sequence alignment in chronological order. In addition, mutations in the S gene during the evolution of the virus strain may be identified. Based on the previous literature and this study, some S-protein mutation sites were identified that may affect tissue tropism [2,3,9,28,29,31]. However, further verification may require a reverse genetics system or the construction of a pseudovirus system.

## 4. Potential Cross-Species Transmission of BCoV

**Figure 4:**
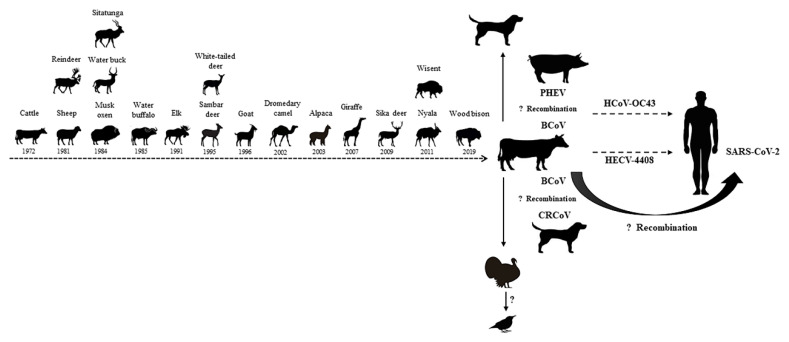
Potential cross-species transmission of BCoV.

Although previous studies have suggested that BCoV maintains genetic stability [84], evidence of BCoV infection in wild animals and children has shown that BCoV has the ability to infect multiple hosts [3,4]. Previous studies have demonstrated that SARS-CoV, which belongs to the same genus (*Betacoronavirus*), is likely to infect humans via an unknown intermediate animal species [87]. Therefore, the appearance of the SARS coronavirus is a warning of crossing the species barrier. BCoV has proven to be capable of infecting and spreading in many animals, including sheep, musk, oxen, elk, sambar, deer, goat, dromedary, camel, alpaca, giraffe, and wisent [4,79,80]. In addition, a molecular clock analysis of BCoV and human coronavirus OC43 (HCoV-OC43) suggests a relatively recent zoonotic transmission event and dates the most recent common ancestor to around 1890 [88]. Moreover, it has recently been reported that OC43 was observed in chimpanzees, indicating that *β-coronaviruses* may undergo recurrent interspecies transmission [89]. In the study conducted by Zhang et al. (1994), the authors isolated the bovine-like coronaviruses strain, HECV-4408, from a case of acute diarrhea in children, and both the nucleotide and amino acid homology between wild-type BCV-ly138 and HECV-4408 were observed to be over 99% [3]. The above studies confirm that BCoV is of great significance in the field of cross-species transmission and have biosafety implications (Figure 4).

Coronaviruses exhibit mutagenic properties and are particularly adept at adapting to new hosts, which was found to be due in part to their substantial capacity for genome recombination [90]. The recombination trend of BCoV was confirmed by analyzing available whole genome sequences, with 51% of sequences displaying evidence of potential recombination events [84]. Keha et al. (2019) demonstrated that a BCoV strain that carried a recombinant HE gene had spread among dairy calves in China [11]. The frequent recombination events of BCoV may be the molecular basis for its changes in tissue tropism and host specificity. Previous studies have found that recombination events may have also occurred between BCoV and other coronaviruses. For example, canine respiratory coronavirus-k37 (CRCoV-k37) may be derived from the genetic recombination between BCoV and CRCoV-BJ232 [91], whereas the OC43 strain, which is closely related to BCoV, may be derived from a recombination event between BCoV and porcine hemagglutinating encephalomyelitis virus. The occurrence of a 290-nucleotide deletion (corresponding to the absence of BCoV ns4.9 and ns4.8) in HCoV-OC43 relative to the BCoV genome potentially supports the argument that an interspecies transmission event occurred from bovines to humans [88]. In addition, genetic recombination and viral mutations, especially gene recombination and mutations in the S protein, can promote the expansion of the host range [92]. In this study, using a sequence alignment analysis, no significant landmark differences were found in the amino acid sequences of the S protein between BCoV and bovine-like coronaviruses. However, a selection pressure analysis revealed that S protein amino acid sites 113, 499, 501, 509, and 1238 were under positive selection (*p* < 0.05) in MEME, SLAC, and FUBAR (Table 2). Despite the fact that no studies have confirmed whether these sites determine the tissue tropism and cross-species transmission of BCoV, further study is deserved based on its high number of mutations.

## 5. Conclusions and Future Perspective

BCoV was first prevalent in America, after which it subsequently broke out in Europe and Asia. In recent years, there have been epidemic reports of BCoV in countries in Oceania and Africa. BCoV has a wide epidemic range and can also cause dual clinical symptoms in the intestine and respiratory tract, resulting in serious economic losses to the global cattle industry. Moreover, in addition to causing bovine infection, bovine-like coronaviruses have been identified in nearly 20 species of animals or humans, indicating that BCoV has the potential for cross-species transmission. Therefore, the epidemic situation, genetic evolution, virus invasion, and other pathogenic mechanisms of BCoV must be urgently clarified. In addition, BCoV shows many common characteristics with SARS-CoV-2. For example, infections with these two viruses may present with similar clinical symptoms, including diarrhea and pneumonia. The common characteristics make it a potentially important reference virus for human coronavirus research, worthy of further study.

### 5.1. Potential Two-Receptor Binding Motif System for BCoV Entry

Viral entry of coronaviruses relies on specific interaction between the S-trimer on the virion surface and a host cell receptor [93]. The S ectodomain comprises a viral attachment, entry subunit S1, and membrane-fusion subunit S2. The S1 subunit contains an N-terminal domain (S1-NTD) that plays a key role in attachment to host cell surface glycans, and a C-terminal domain (S1-CTD) with a receptor-binding domain (RBD) responsible for specific binding to a host protein receptor. S1-CTDs are stabilized in an inactive “lying-down” conformation and expand into the active “standing-up” conformation once the S-trimer engages with the host receptor. Host receptor engagement destabilizes the S-trimer, exposing the cleavage site between S1 and S2 subunits, which is then cleaved by a cathepsin, TMPRSS2, or another extracellular protease to initiate S2-mediated membrane fusion and viral entry [94].

The host receptor-binding S1 subunit, especially S1-NTD and S1-CTD/RBD, displays marked variation among coronaviruses and is the primary determinant of host tropism and transmission limits [93]. To date, it is proposed that coronaviruses may use the two-receptor binding motif (RBM) system [94]. For most coronaviruses, the N-terminal domain (NTD) of the S1 subunit recognizes cell-surface carbohydrates, while the C-terminal domain (CTD) binds specifically to cellular protein receptors [95]. Previous reports have shown that BCoV uses 5-N-acetyl-9-O-acetylneuraminic acid (Neu5, 9Ac2) as a receptor recognized by S1-NTD [85]; however, recent studies suggest that Neu5, 9Ac2 may only be the attachment receptor for BCoV. The study by Bidokhti et al. (2013) proposed that the SI-CTD (amino acids 326 to 540) of the BCoV strain contained a putative receptor-binding domain that may bind to specific protein receptors [90]. In addition, Szczepanski et al. (2019) blocked the interaction between the virus and HLA-I molecules using polyclonal antibodies, which blocked infection by BCoV, suggesting that HLA-I serves as an entry receptor for BCoV [96]. However, its binding position and further receptor verification has not been reported. In addition, in preliminary experiments involving OC43, which is closely related to BCoV, the OC43 virus was shown to have affinity with HLA class I antigen. Thus, the HLA class 1 antigen may serve as a receptor for human coronavirus OC43 [81]. Moreover, it has been reported that HCoV-HKU1, which belongs to same subgenus (subgenus *Embecovirus*, genus *Betacoronavirus*), may use HLA-I as the attachment factor [97] and its receptor-binding domain is located in S1-CTD [98]. Based on the present literature, we speculate that BCoV may also use the two-receptor binding motif (RBM) system, in which using Neu5, 9Ac2 acts as a glycan attachment receptor recognized by S1-NTD, and using HLA-I as a protein attachment receptor recognized by S1-CTD.

Millet et al. (2021) suggests that, in some ways, coronavirus S1 can be considered to have evolved the NTD- and CTD-binding modules as a means to broaden its cell tropism within a host, as well as between different host species. Perhaps the NTD may play a critical role during species barrier-crossing events by allowing an emerging coronavirus to adapt to a new host environment and maintain a minimal level of binding to allow the infection of new host cells via sialic acids. In contrast, the CTD readjusts and gains adaptive mutations for optimizing binding to a new host protein receptor [99]. Therefore, we also speculate that the putative dual-receptor recognition system may play an important role in potential cross-species transmission of BCoV. To date, the cellular receptor for BCoV is uncertain and the mechanism of viral entry is unclear, limiting the research involving viral tropism and potential cross-species transmission. In recent years, pseudovirus has been extensively explored in viral research, antiviral screening, and receptor-binding assays. Constructing S-NTD and S-CTD using pseudoviruses and evaluating their binding efficiency with putative receptors may be an effective method to verify the double-receptor recognition system and explore BCoV invasion, which warrants further investigation.

### 5.2. BCoV and SARS-CoV-2

As members of the *Coronaviridae* β genus, BCoV and SARS-CoV-2 share some common pathogenic characteristics. Their interspecies transmission and other factors that affect the severity of bovine disease parallel that of SARS-CoV-2 [1]. Therefore, these viruses may have comparable pathogenesis and transmission characteristics.

SARS-CoV-2 has newly emerged and spreads epidemically, whereas BCoV has existed for long periods, is endemic worldwide, and relatively well-known. Given that both pneumonia and diarrhea occur in COVID-19 patients and BCoV-infected cattle, knowledge about the pathogenesis and transmission characteristics of BCoV may contribute to a better understanding of SARS-CoV-2. For BCoV infection, direct contact and indirect transmission by fomites are the most likely route of transmission between herds; however, there were indications of airborne transmission during an experimental infection. Although the persistence of SARS-CoV-2 on various materials is low [100], the risk for indirect transmission via fomites should not be neglected. In addition, several studies have proposed that BCoV may be used as a model to study SARS-CoV-2; the use of BCoV as a model for human coronavirus, including SARS-CoV-2, is feasible and advantageous. Especially in experimental animals, cattle are relatively easy to obtain, and can provide a unique model for comparative immunology research. Indeed, most immunological signaling systems are evolutionarily conserved in mammals [101] and the majority of immune cell phenotypes and functional characteristics exhibit distinct similarities with humans [102]. In terms of prevention and control measures, as a possible control measure against SARS-CoV-2, Arenas et al. (2021) proposed the use of cow’s milk from animals immune to BCoV [103]. A recent study revealed that cattle were susceptible to SARS-CoV-2 infection. Therefore, dual infections in individual animals might lead to recombination events between SARS-CoV-2 and BCoV [104]. Therefore, the further study of BCoV is of great significance. First, it may be useful for enhancing our understanding of the mechanisms of disease related to SARS-CoV-2 infection and the potential host factors that cause SARS-CoV-2 severity. Second, since dual infections of individual animals might lead to recombination events between SARS-CoV-2 and BCoV, this may also severely affect human biosafety. Thus, future long-term epidemiological surveys considering BCoVs/bovine-like coronaviruses from birds, cattle, other animals, and humans are required.

## Figures and Tables

**Table 1:** The transmission of BCoV.

Sample Source	Time Sequence	Continent	Country	Positive Rate	References
Intestinal tract	Before 2000	America	USA	16.4–84%	[21,22]
			Canada	6.5–70%	[9,19,23]
			Argentina	2.41–10.52%	[20,46]
		Europe	Britain	14%	[24]
			Belgium	8%	[25]
		Asia	Japan	14.5–62.5%	[10,26]
	2000–2009	America	Brazil	14.91–68.6%	[35,36]
			Uruguay	1.5–11.8%	[47]
		Europe	Netherlands	2.8%	[7]
			Italy	46.74%	[33]
		Asia	Korea	5.6–58.2%	[27,30,31,32]
			Turkey	10.8–28.1%	[28,29]
	2010–2019	Oceania	Australia	21.6%	[39]
			New Zealand	14%	[38]
		Africa	Algeria	20.73%	[40]
			Ghana	0.3%	[8]
		Asia	Iran	7.2%	[41]
			China	12.20–69.05%	[11,45]
			Thailand	12%	[42]
			India	8.88–16%	[43]
			Vietnam	6.9%	[44]
Respiratory tract	Before 2000	America	USA	8.1–96%	[6,48]
			Canada	57.89–66.67%	[23,49]
	2000–2009	Europe	Italy	9.6–65.85%	[50]
	2010–2019	America	Brazil	22–67%	[35,37]
		Europe	Ireland	22.9–60.7%	[51]
			France	17–70.1%	[52,53]
		Asia	Japan	21.2%	[54]
			Turkey	1%	[28]
		Oceania	Australia	13–33.33%	[55]
	After 2020	Asia	China	21.53%	[45]
Serum antibody		America	USA	11–91%	[22]
			Canada	7–100%	[56,57]
		Europe	Sweden	23.8–100.0%	[58]
			Norway	16–72.2%	[59,60,61]
			France	16.5%	[62]
			Belgium	30%	[63]
		Africa	Ghana	55.8%	[8]

**Table 2:** Amino acid sites detected to be under positive selection (with *p*-values < 0.05) through three election methods (MEME, SLAC, and FUBAR).

Codon	MEME *p*-Value	SLAC *p*-Value	FUBAR *p*-Value
113	0.020	0.015	0.000
499	0.01	0.007	0.000
501	0.000	0.000	0.000
509	0.000	0.003	0.000
1238	0.000	0.028	0.041

## Data Availability

Data sharing not applicable.

## Supplementary Materials

The following supporting information can be downloaded at: https://www.mdpi.com/article/10.3390/v14051109/s1, Table S1: The reference BCoV strains used in this study.
