# Supplementary material for: Advances in Bovine Coronavirus Epidemiology
Source: Viruses. 2022 May 21;14(5):1109. doi: 10.3390/v14051109 (PMC9147158; doi:10.3390/v14051109)
Supplement: Supplementary file 1 [file viruses-14-01109-s001.zip › viruses-1702708-supplementary.pdf]

Table S1 The reference BCoV strains used in this study.

Strain name	Countries	Collection date	GenBank accession no.
Kakegawa	Japan	1976	AB354579.1
LY-138	USA	1965	AF058942.1
LSU-94LSS-051-2	USA	1994	AF058943.1
OK-0514-3	USA	1996	AF058944.1
Quebec	Canada	1972	AF220295.1
BCoV-ENT	USA	1998	AF391541.1
BCoV-LUN	USA	1998	AF391542.1
KWD1	Korea	2002	AY935637.1
KWD2	Korea	2002	AY935638.1
KWD3	Korea	2002	AY935639.1
KWD4	Korea	2002	AY935640.1
KWD5	Korea	2002	AY935641.1
KWD6	Korea	2002	AY935642.1
KWD7	Korea	2002	AY935643.1
KWD8	Korea	2002	AY935644.1
KWD9	Korea	2002	AY935645.1
KWD10	Korea	2002	AY935646.1
F15	France	1979	D00731.1
KCD1	Korea	2004	DQ389632.1
KCD2	Korea	2004	DQ389633.1
KCD3	Korea	2004	DQ389634.1
KCD4	Korea	2004	DQ389635.1
KCD5	Korea	2004	DQ389636.1
KCD6	Korea	2004	DQ389637.1
KCD7	Korea	2004	DQ389638.1
KCD8	Korea	2004	DQ389639.1
KCD9	Korea	2004	DQ389640.1
KCD10	Korea	2004	DQ389641.1
KWD11	Korea	2002	DQ389652.1
KWD12	Korea	2002	DQ389653.1
KWD13	Korea	2002	DQ389654.1
KWD14	Korea	2002	DQ389655.1
KWD15	Korea	2002	DQ389656.1
KWD16	Korea	2002	DQ389657.1
KWD17	Korea	2002	DQ389658.1
KWD18	Korea	2002	DQ389659.1
KWD19	Korea	2002	DQ389660.1
Alpaca	USA	1998	DQ915164.2
Sable_antelope_coronavirus_US/OH1/2003	USA	2003	EF424621.1
Giraffe_coronavirus_US/OH3/2003	USA	2003	EF424623.1
Calf-giraffe_coronavirus_US/OH3/2006	USA	2006	EF424624.1
Human_enteric_coronavirus_4408	Germany	1988	FJ415324.1
DB2	Germany	1983	DQ811784.2
W17-18/R/2017	Korea	2017	MG518518.1
Waterbuck_coronavirus_US/OH-WD358	USA	1994	FJ425186.1
White-tailed_deer_coronavirus_US/OH-WD470	USA	1994	FJ425187.1
Human_enteric_coronavirus_strain_4408	USA	2009	FJ938067.1
Canine_respiratory_coronavirus_strain_BJ232	China	2014	KX432213.1
E-AH187	USA	2000	EF424619.1
R-AH187	USA	2000	EF424620.1
Bovine coronavirus E-AH65	USA	2007	EF424615.1
339/06	Italy	2006	EF445634.1
Bubalus/Italy/179/07-11	Italy	2007	EU019216.1
438/06-TN	Italy	2006	EU814647.1
438/06-TN-50	Italy	2006	EU814648.1
Wisent 10/01	Korea	2010	HM573326.1

Tahr1 10/01	Korea	2010	HM573327.1
Tahr2 10/01	Korea	2010	HM573328.1
Sitatunga 10/01	Korea	2010	HM573329.1
Nyala 10/01	Korea	2010	HM573330.1
SWE/C/92	Sweden	1992	KF169908.1
SWE/02-1	Sweden	2002	KF169909.1
SWE/02-2	Sweden	2002	KF169910.1
SWE/02-3	Sweden	2002	KF169911.1
SWE/02-4	Sweden	2002	KF169912.1
DEN/03-1	Denmark	2003	KF169913.1
DEN/03-2	Denmark	2003	KF169914.1
DEN/03-3	Denmark	2003	KF169915.1
DEN/05-1	Denmark	2005	KF169916.1
DEN/05-2	Denmark	2005	KF169917.1
DEN/05-3	Denmark	2005	KF169918.1
DEN/05-4	Denmark	2005	KF169919.1
SWE/N/05-1	Sweden	2005	KF169920.1
SWE/N/05-2	Sweden	2005	KF169921.1
SWE/AC/06-1	Sweden	2006	KF169922.1
SWE/M/06-3	Sweden	2006	KF169923.1
SWE/M/06-4	Sweden	2006	KF169924.1
SWE/Z/07-1	Sweden	2007	KF169925.1
SWE/C/07-2	Sweden	2007	KF169926.1
SWE/I/07-3	Sweden	2007	KF169927.1
SWE/I/07-4	Sweden	2007	KF169928.1
SWE/I/07-5	Sweden	2007	KF169929.1
SWE/C/07-6	Sweden	2007	KF169930.1
SWE/AC/08-1	Sweden	2008	KF169931.1
SWE/C/08-2	Sweden	2008	KF169932.1
SWE/I/08-3	Sweden	2008	KF169933.1
SWE/P/09-1	Sweden	2009	KF169934.1
SWE/C/09-2	Sweden	2009	KF169935.1
SWE/U/09-3	Sweden	2009	KF169936.1
SWE/M/10-1	Sweden	2010	KF169937.1
SWE/M/10-2	Sweden	2010	KF169938.1
SWE/Y/10-3	Sweden	2010	KF169939.1
SWE/P/10/4	Sweden	2010	KF169940.1
HLJ-14/CHN	China	2014	KM985631.1
BCoV/FRA/EPI/Caen/2005/01	France	2003	KT318111.1
BCoV/FRA/EPI/Caen/2005/02	France	2005	KT318112.1
BCoV/FRA/EPI/Caen/2008/04	France	2008	KT318114.1
BCoV/FRA/EPI/Caen/2003/05	France	2003	KT318115.1
BCoV/FRA/EPI/Caen/2010/06	France	2010	KT318116.1
BCoV/FRA/EPI/Caen/2012/07	France	2012	KT318117.1
BCoV/FRA/EPI/Caen/2013/08	France	2013	KT318118.1
BCoV/FRA/EPI/Caen/2013/09	France	2013	KT318119.1
BCoV/FRA/EPI/Caen/2013/10	France	2013	KT318120.1
BCoV/FRA/EPI/Caen/2013/11	France	2013	KT318121.1
BCoV/FRA/EPI/Caen/2014/12	France	2014	KT318122.1
BCoV/FRA/EPI/Caen/2014/13	France	2014	KT318123.1
BCoV/FRA/EPI/Caen/2004/14	France	2004	KT318124.1
BCV-AKS-01	China	2015	KU886219.1
BCoV_2014_13	France	2014	KX982264.1
GIF-1	Japan	2016	LC494126.1
IWT-9	Japan	2012	LC494127.1
IWT-10	Japan	2012	LC494128.1
IWT-1	Japan	2010	LC494129.1
IWT-2	Japan	2010	LC494130.1
IWT-3	Japan	2011	LC494131.1
IWT-4	Japan	2011	LC494132.1
IWT-5	Japan	2011	LC494133.1

IWT-6	Japan	2011	LC494134.1
IWT-7	Japan	2012	LC494135.1
IWT-8	Japan	2012	LC494136.1
IWT-11	Japan	2013	LC494137.1
IWT-12	Japan	2015	LC494138.1
IWT-13	Japan	2015	LC494139.1
IWT-14	Japan	2015	LC494140.1
IWT-15	Japan	2016	LC494141.1
IWT-18	Japan	2016	LC494142.1
IWT-19	Japan	2016	LC494143.1
IWT-20	Japan	2016	LC494144.1
IWT-21	Japan	2016	LC494145.1
IWT-16	Japan	2016	LC494146.1
IWT-17	Japan	2016	LC494147.1
IWT-22	Japan	2016	LC494148.1
IWT-23	Japan	2016	LC494149.1
IWT-24	Japan	2017	LC494150.1
IWT-25	Japan	2017	LC494151.1
IWT-26	Japan	2017	LC494152.1
IWT-27	Japan	2017	LC494153.1
SHG-1	Japan	2014	LC494154.1
SHG-2	Japan	2014	LC494155.1
SHG-3	Japan	2014	LC494156.1
SHG-4	Japan	2015	LC494157.1
SHG-5	Japan	2016	LC494158.1
SHG-6	Japan	2017	LC494159.1
TCG-4	Japan	2007	LC494160.1
TCG-5	Japan	2007	LC494161.1
TCG-3	Japan	2006	LC494162.1
TCG-2	Japan	2006	LC494163.1
TCG-1	Japan	2006	LC494164.1
TCG-10	Japan	2008	LC494165.1
TCG-11	Japan	2008	LC494166.1
TCG-12	Japan	2008	LC494167.1
TCG-13	Japan	2009	LC494168.1
TCG-14	Japan	2009	LC494169.1
TCG-15	Japan	2010	LC494170.1
TCG-16	Japan	2010	LC494171.1
TCG-6	Japan	2007	LC494172.1
TCG-7	Japan	2008	LC494173.1
TCG-8	Japan	2008	LC494174.1
TCG-18	Japan	2016	LC494175.1
TCG-17	Japan	2016	LC494176.1
TCG-9	Japan	2008	LC494177.1
TCG-19	Japan	2016	LC494178.1
TCG-20	Japan	2016	LC494179.1
TCG-21	Japan	2016	LC494180.1
TCG-22	Japan	2016	LC494181.1
TCG-23	Japan	2016	LC494182.1
TCG-24	Japan	2017	LC494183.1
TCG-25	Japan	2017	LC494184.1
TCG-26	Japan	2017	LC494185.1
TCG-27	Japan	2017	LC494186.1
TCG-28	Japan	2017	LC494187.1
TCG-29	Japan	2017	LC494188.1
TCG-30	Japan	2017	LC494189.1
TCG-31	Japan	2017	LC494190.1
TCG-32	Japan	2017	LC494191.1
TCG-33	Japan	2017	LC494192.1
ICSA21-LBA	France	2014	MG757138.1
ICSA16-EN	France	2014	MG757139.1

ICSA16-LBA	France	2014	MG757140.1
ICSA-pool-EN	France	2014	MG757141.1
ICSA-pool-LBA	France	2014	MG757142.1
ICSA4-EN	France	2014	MG757143.1
ICSA17-LBA	France	2014	MG757144.1
4-17-03	USA	2017	MH043952.1
4-17-25	USA	2017	MH043953.1
4-17-08	USA	2017	MH043954.1
7-16-23	USA	2016	MH043955.1
PL84	Vietnam	2017	MH197037.1
PL83	Vietnam	2017	MH197038.1
ND65	Vietnam	2017	MH197039.1
DT97	Vietnam	2017	MH203064.1
BL104	Vietnam	2017	MH203065.1
MC199	Vietnam	2017	MH203066.1
VP200	Vietnam	2017	MH203067.1
Yak/HY24	China	2017	MH741424.1
QH1	China	2017	MH810151.1
QH2	China	2017	MH810152.1
QH3	China	2017	MH810153.1
SC1	China	2017	MH810154.1
SC2	China	2017	MH810155.1
SC3	China	2017	MH810156.1
XZ1	China	2017	MH810157.1
XZ2	China	2017	MH810158.1
XZ3	China	2017	MH810159.1
YN1	China	2017	MH810160.1
YN2	China	2017	MH810161.1
YN3	China	2017	MH810162.1
HY24	China	2017	MH810163.1
BCOV-China/SWUN/SC1/2017	China	2017	MK095174.1
BCOV-China/SWUN/SC2/2017	China	2017	MK095175.1
BCOV-China/SWUN/SC3/2017	China	2017	MK095176.1
BCOV-China/SWUN/SX1/2018	China	2018	MK095177.1
BCOV-China/SWUN/SX2/2018	China	2018	MK095178.1
BCOV-China/SWUN/LN1/2018	China	2018	MK095179.1
BCOV-China/SWUN/LN2/2018	China	2018	MK095180.1
BCOV-China/SWUN/LN3/2018	China	2018	MK095181.1
BCOV-China/SWUN/LN4/2018	China	2018	MK095182.1
BCOV-China/SWUN/LN5/2018	China	2018	MK095183.1
BCOV-China/SWUN/HN1/2018	China	2018	MK095184.1
BCOV-China/SWUN/HN2/2018	China	2018	MK095185.1
BCOV-China/SWUN/HN3/2018	China	2018	MK095186.1
BCOV-China/SWUN/A10/2018	China	2018	MN982199.1
BCOV-China/SWUN/A1/2018	China	2018	MN982198.1/ /
4950	Turkey	2016	MK989620.1
4945	Turkey	2016	MK989621.1
U1	Turkey	2016	MK989622.1
T5	Turkey	2018	MK989623.1
S8	Turkey	2017	MK989624.1
Mebus	USA	1972	U00735.2
R-AH65	USA	2000	EF424617.1
V270	Germany	1983	EF193075.1
AH187	USA	2000	FJ938065.1
